# Do Dogs and Cats Passively Carry SARS-CoV-2 on Hair and Pads?

**DOI:** 10.3390/v13071357

**Published:** 2021-07-13

**Authors:** Stefania Lauzi, Angelica Stranieri, Alessia Giordano, Davide Lelli, Gabriella Elia, Costantina Desario, Gabriele Ratti, Nicola Decaro, Saverio Paltrinieri

**Affiliations:** 1 Department of Veterinary Medicine, University of Milan, 26900 Lodi, Italy; stefania.lauzi@unimi.it (S.L.); angelica.stranieri@unimi.it (A.S.); gabriele.ratti@unimi.it (G.R.); saverio.paltrinieri@unimi.it (S.P.); 2 Istituto Zooprofilattico Sperimentale della Lombardia e dell’Emilia-Romagna “Bruno Ubertini”, 25124 Brescia, Italy; davide.lelli@izsler.it; 3 Department of Veterinary Medicine, University of Bari “Aldo Moro”, Valenzano, 70010 Bari, Italy; gabriella.elia@uniba.it (G.E.); costantina.desario@uniba.it (C.D.); nicola.decaro@uniba.it (N.D.)

**Keywords:** SARS-CoV-2, skin, hair, dog, cat, COVID-19 patients

## Abstract

The epidemiological role of domestic animals in the spread and transmission of SARS-CoV-2 to humans has been investigated in recent reports, but some aspects need to be further clarified. To date, only in rare cases have dogs and cats living with COVID-19 patients been found to harbour SARS-CoV-2, with no evidence of pet-to-human transmission. The aim of the present study was to verify whether dogs and cats act as passive mechanical carriers of SARS-CoV-2 when they live in close contact with COVID-19 patients. Cutaneous and interdigital swabs collected from 48 dogs and 15 cats owned by COVID-19 patients were tested for SARS-CoV-2 by qRT-PCR. The time elapsed between owner swab positivity and sample collection from pets ranged from 1 to 72 days, with a median time of 23 days for dogs and 39 days for cats. All samples tested negative, suggesting that pets do not passively carry SARS-CoV-2 on their hair and pads, and thus they likely do not play an important role in the virus transmission to humans. This data may contribute to confirming that the direct contact with the hair and pads of pets does not represent a route for the transmission of SARS-CoV-2.

## 1. Introduction

The current pandemic of coronavirus disease 2019 (COVID-19) has caused over two and a half million human deaths (as of 15 March 2021) and is being sustained by the human-to-human transmission of severe acute respiratory syndrome coronavirus-2 (SARS-CoV-2) [1].

Although COVID-19 is primarily transmitted from person to person via respiratory droplets, and faecal–oral transmission may also occur [1,2], contamination via surfaces has also been suggested as a potential form of transmission of SARS-CoV-2 [3]. SARS-CoV-2 may be viable on environmental surfaces for up to 72 h under laboratory conditions, and viral load and temperature have been shown to influence the presence of infectious virus on common surfaces for up to 28 days [4].

Moreover, the detection of SARS-CoV-2 in pets raised questions about the possible zoonotic transmission from cats and dogs in close contact with humans. During the first months of the pandemic, official Italian websites included answers for pet owners who frequently asked if the pads of their pets could be contaminated by surfaces after a walk and if pads had to be cleaned when returning home in order to prevent the spread of COVID-19 [5] (Italian Ministry of Health, 2021).

Currently, SARS-CoV-2 infections are rarely detected in pets, and almost all SARS-CoV-2-positive pets identified so far belonged to COVID-19 positive owners, suggesting human-to-pet transmission [6,7,8]. On the contrary, to date, there is no evidence of pet-to-human transmission, suggesting that pets, even if infected, do not play an epidemiological role in the transmission of SARS-CoV-2 to their owners. Further investigation is needed to assess the potential role of pets in the spread of SARS-CoV-2 [9]. To the best of our knowledge, the data on SARS-CoV-2 contamination of hair and pads of pets are limited and mostly refer to dogs or cats infected by SARS-CoV-2, as demonstrated by PCR positive oropharyngeal, nasal, and/or rectal swabs [10,11,12]. Therefore, the aim of this study was to assess whether hair and pads of pets from COVID-19 positive households are contaminated by their owner(s) and/or indirectly by the environment in order to determine if pets, even if they are not actively infected, may passively carry the virus.

## 2. Materials and Methods

The study included 48 dogs and 15 cats from Italian COVID-19-positive owners that were diagnosed in April–May and October–November 2020 with COVID-19 on the basis of the presence of symptoms and signs consistent with the disease [13] and a positive nasopharyngeal swab for SARS-CoV-2, as officially defined by the National Public Health services. The inclusion criteria for dogs and cats were the absence of clinical signs and, in order to exclude self-contamination, the absence of SARS-CoV-2 RNA in nasal, oropharyngeal, and rectal swabs of the animals, determined as previously described [14].

Samples from dogs and cats were collected during routine veterinary visits under the informed consent of the owners, who also filled out a questionnaire including information about the date of their clinical and molecular diagnosis. Dogs and cats were brought to the veterinary visit by their owners (after the owners had recovered and received negative results of nasopharyngeal swabs) or, if veterinary visits were requested before the recovery of the owners, by healthy dog sitters or relatives not living with the owners. In four cases, animals were owned by veterinarians, who directly performed the samplings following the authors’ instructions. The study was approved by the Institutional Animal Care and Use Committee and by the Institutional Ethical Committee (approval numbers_31/20 and 43/20, respectively).

Cutaneous samples from dogs and cats were collected by brushing sterile swabs on the skin and hair of the dorsal part of the neck and moving the swab towards the lumbar region. To collect interdigital samples, swabs were brushed on the surface of the footpads and then gently inserted into the interdigital spaces. These anatomical regions were selected based on the frequency of direct contact between pets and owners (dorsal part of animal bodies) and between pets and the environment (footpads and interdigital spaces). When delivered to the Veterinary Teaching Hospital (VTH) of the University of Milan, each swab was dipped in a solution based on phosphate-buffered saline (PBS pH 7.2) supplemented with 10% glycerol and antibiotics (1%) and frozen at −20 °C until shipping in cold chain to the Istituto Zooprofilattico Sperimentale della Lombardia e dell’Emilia Romagna for analyses, which are described below. Swabs taken by veterinarians were frozen and then delivered to the VTH after the veterinarians’ recovery and negative testing, then treated as described above.

Swabs were thawed and centrifuged at 3750 rpm for 15 min. Viral RNA was extracted from 250 µL of supernatant using the QIAsymphony™ SP instrument (Qiagen, Hilden, Germany) according to the manufacturer’s instructions. Swabs from dogs and cats were analysed by real-time reverse transcription-PCR (qRT-PCR) tests targeting a specific region of the SARS-CoV-2 E gene to detect SARS-CoV-2 as previously described [15]. Thermal cycling was performed at 52 °C for 15 min for reverse transcription, followed by 95 °C for 10 s and then 45 cycles of 95 °C for 5 s and 60 °C for 30 s.

In our study, the analytical sensitivity (LOD) of the assay was 11–15 copies of SARS-COV-2 RNA per reaction at a 99% detection probability in human respiratory specimens. Multiple negative controls were included in each run to rule out contamination, while an internal positive control (IC) was added to each sample in order to reveal the presence of RT-PCR inhibitors. ICs were simultaneously extracted and/or amplified in the same tube with the pathogen target; combined with the amplification positive control, they proved the functionality of the reaction mixture for correct amplification of the SARS-COV-2 E gene target. This combination ruled out inhibition and other malfunctions and confirmed that negative results were truly negative. The amplification positive control consisted of SARS-CoV-2 RNA extracts from swabs of infected human patients, diluted until a CT value (cycle threshold) of 26–30 was obtained.

## 3. Results and Discussion

**Figure 1:**
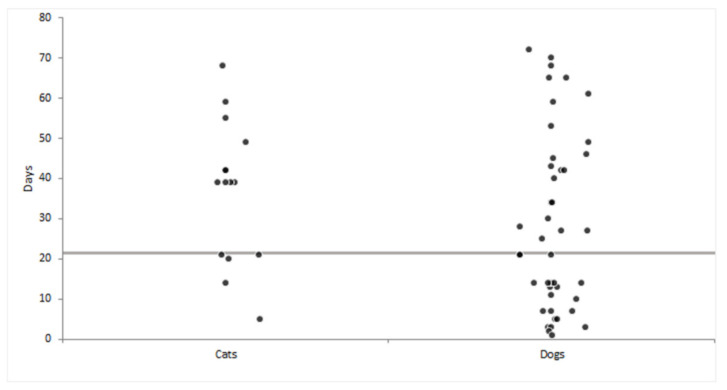
Time elapsed from the detection of SARS-CoV-2 in owners by oropharyngeal swab and the collection of swabs of hair and footpads from dogs and cats. The grey horizontal line indicates 21 days from the owner’s positive test.

The time elapsed from the positive testing of owners and the collection of swabs from pets ranged from 1 to 72 days (median 23 days) in dogs and from 5 to 68 days (median 39 days) in cats (Figure 1). Specifically, of the 63 samples, 42.9% (*n* = 27) were collected within 21 days of the owner’s positive test and comprised 22 swabs from dogs and 5 from cats, whereas 57.1% of samples (*n* = 36) were collected between 22 and 72 days after the owner’s positive test and comprised 26 swabs from dogs and 10 from cats.

All cutaneous and interdigital swabs tested negative for SARS-CoV 2 RNA.

The current guidelines of the World Organisation for Animal Health (OIE) recommend that owners infected with SARS-CoV-2 avoid close contact with their companion animals [16]. However, the complete absence of contact or indirect contamination of pets through environmental surfaces/fomites (e.g., through the owners’ coughing or sneezing) is unlikely in pets in COVID-19-positive households. The results of the present study suggest that pets are not contaminated by their owners or the environment or that routine hygiene practices are adequate to avoid persistent contamination of pets’ hair and pads with SARS-CoV-2. The time elapsed from the owner’s COVID-19 diagnosis to pet sample collection is considered a potential source of bias for some of our negative results [17]. It was reported that SARS-CoV-2 in humans is contagious for several days after onset of symptoms, and some patients test positive for SARS-CoV-2 RNA for weeks if not months [16]. Contagiousness in patients rapidly decreases after about 10–15 days from the onset of COVID-19, depending on the severity of the clinical presentation and whether the patients are immunocompromised. The longest duration of viral viability that has been reported thus far is 20 days from the onset of clinical signs [18]. Moreover, the viral load and temperature were shown to influence the presence of infectious virus on common surfaces for up to 28 days [4]. Although it has been demonstrated that the probability of pets being seropositive increases with the time of exposure to the infected owner [19], it may be possible that the virus was present on the hair or pads of pets before they were sampled. This is in line with a recent report showing that of 56 dogs with negative oropharyngeal and rectal swabs who lived with SARS-CoV-2 positive owners, 1 tested positive in a fur sample but tested negative soon after [12]. Nevertheless, negative results were also observed in our study in samples from pets that were collected within 21 days after the diagnosis of COVID-19 in their owners, when it was likely that symptomatic people or housemates still harboured the virus. These results suggest that pets do not play an epidemiological role in the spread and indirect transmission of SARS-CoV-2 to humans via contamination of hair or paws.

The limitations of this study include the low number of enrolled animals (due to the challenges of collecting samples from pets belonging to COVID-19 patients) and the possible influence of preanalytical or analytical factors. Among these, the specificity and sensitivity of the method seem not to be an issue based on the results of our own evaluation of the analytical performances of the method and on the use of positive and negative controls. This evaluation did not include the assessment of possible storage artefacts. Although storage at −20 °C may have theoretically affected sensitivity especially if the virus load in skin or pad swabs was low, previous reports have demonstrated limited pre-analytical artefacts in samples stored at room temperature, +4 °C, or −20 °C, and it is therefore unlikely that freezing conditions led to false-negative results [20,21]. Despite these limitations, our results suggest that pets do not passively carry the virus or play an important role in the transmission of SARS-CoV-2 to humans through direct contact with the hair or skin of pets. Considering the data obtained in this study, future investigations using a One Health approach are needed to update the knowledge of the possible risk of pet-to-human transmission in the early phase of infection. Moreover, future studies are recommended to evaluate whether owners’ behaviors and interactions with their pets influence the likelihood that their pet will test positive or have positive hair or paw swabs in order to design effective strategies for COVID-19 prevention and control and to avoid unnecessary discrimination against animals.

## Data Availability

The data presented in this study are available on request from the corresponding author.
